# PVRI‐GoDeep—A Global Meta‐Registry at the Crossroads of Heart and Lung

**DOI:** 10.1002/cph4.70118

**Published:** 2026-03-24

**Authors:** Meike T. Fuenderich, Athiththan Yogeswaran, Patrick Janetzko, Khodr Tello, Werner Seeger, Raphael W. Majeed, Jochen Wilhelm, GoDeep Consortium, Mauro Acquaro, Imad Al Ghouleh, James Anderson, Jeffrey S. Annis, Anastasia Anthi, Alexandra Arvanitaki, Aparna Balasubramanian, Felix Ballmann, Harm Jan Bogaard, Evan Brittain, Hugh Buzacott, Hector R. Cajigas, John Cannon, Stephen Y. Chan, Victoria Damonte, Effrosyni Dima, Philipp Douschan, Nathan Dwyer, Diego Echazarreta, Christina A. Eichstaedt, Jean Elwing, Kai Förster, Robert Frantz, Marlize Frauendorf, Stefano Ghio, Hossein‐Ardeschir Ghofrani, George Giannakoulas, Friedrich Grimminger, Ekkehard Grünig, Lars Harbaum, Paul M. Hassoun, Melanie Heberling, Anne Hilgendorff, Luke Howard, Arun Jose, Ernesto Junaeda, David G. Kiely, Ingrid King, Hans Klose, Ziad Konswa, Gabor Kovacs, Philipp Krieb, Keiichiro Kuronuma, Edmund M. Lau, Melanie Lavender, Allan Lawrie, Mona Lichtblau, Kurt Marquardt, Hiromi Matsubara, Farhan Mubashir, Horst Olschewski, Mauricio Orozco‐Levi, Karen Osborn, Joanna Pepke‐Zaba, Alba Ramirez‐Sarmiento, Stephan Rosenkranz, Hani Sabbour, Sandeep Sahay, Khaled Saleh, Laura Scelsi, Yuriy Sirenko, Siva Sivakumaran, Andrew J. Sweatt, Thenappan Thenappan, Ioan Tilea, Olena Torbas, Silvia Ulrich, Andrea Varga, Helen M. Whitford, Christoph B. Wiedenroth, Martin R. Wilkins, Paul G. Williams, Shaun Yo, Roham T. Zamanian, Zhenguo Zhai, Zhu Zhang

**Affiliations:** ^1^ Department of Internal Medicine Universities of Giessen and Marburg Lung Center (UGMLC), Member of the German Center for Lung Research (DZL) Giessen Germany; ^2^ Institute for Lung Health (ILH), Cardio‐Pulmonary Institute (CPI) Giessen Germany; ^3^ Institute of Medical Informatics RWTH Aachen University Aachen Germany

**Keywords:** data integration, data quality management, meta‐registry, pulmonary hypertension, real world evidence, right heart failure

## Abstract

Pulmonary hypertension (PH) is a complex disease characterized by increased pressure in the pulmonary arteries. It encompasses a heterogeneous group of entities that increase right heart afterload and often lead to right heart failure and premature death. Advancing diagnosis, risk stratification, and treatment across the diverse PH spectrum requires large, high‐quality, longitudinal datasets that exceed the scope of individual national or regional registries. To address this need, PVRI GoDeep was founded under the umbrella of the Pulmonary Vascular Research Institute (PVRI). This global meta registry harmonizes and integrates anonymized patient‐level data from existing PH registries at expert centers worldwide. PVRI GoDeep enables the reuse of locally collected real‐world data by mapping heterogeneous datasets to a predefined data dictionary, thorough quality checks, and regular updates. This approach supports phenotyping across all PH groups, enables international comparisons, and allows in‐depth analysis of rare subtypes, disease progression, treatment responses, and survival rates. Importantly, GoDeep makes clinically relevant research possible that cannot be conducted at the level of a single center, such as validating risk‐stratification tools across PH subgroups, evaluating off‐label therapies, and investigating newly recognized entities, such as mild PH. By January 2026, data from more than 45,000 individuals worldwide were integrated into GoDeep, making it one of the largest and most diverse PH registries. Offering a scalable, governed, and disease‐independent framework for harmonized real‐world evidence generation, PVRI GoDeep is a powerful platform to deepen the understanding of PH and support the development of clinical guidelines for the diagnosis and treatment of PH.

## Graphical abstract

PVRI GoDeep is a global meta‐registry that harmonizes high‐quality patient‐level data from pulmonary hypertension registries worldwide. With over 45,000 enrolees already included, it enables robust phenotyping, international comparisons, and clinically relevant research across all PH subgroups beyond the scope of single‐center or national datasets.

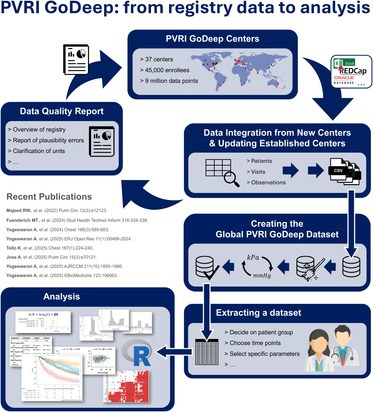

## 1 Introduction

Pulmonary Hypertension (PH) is a complex disorder defined by elevated pressure in the pulmonary arteries, a condition that can lead to right heart failure and premature death. It represents a heterogeneous spectrum of diseases with multiple underlying causes and is categorized into five clinical groups, including pulmonary arterial hypertension (PAH), PH due to left heart disease, and PH associated with lung disorders, among others (Humbert et al. 2022). All of these groups are further sub‐divided into specific categories, as shown in Table 1.

**TABLE 1:** Clinically defined subtypes of pulmonary hypertension (Humbert et al. 2022).

Pulmonary hypertension classification
GROUP 1 Pulmonary arterial hypertension (PAH)*
1.1 Idiopathic
1.1.1 Non‐responders at vasoreactivity testing
1.1.2 Acute responders at vasoreactivity testing
1.2 Heritable
1.3 Associated with drugs and toxins
1.4 Associated with:
1.4.1 Connective tissue disease
1.4.2 HIV infection
1.4.3 Portal hypertension
1.4.4 Congenital heart disease
1.4.5 Schistosomiasis
1.5 PAH with features of venous/capillary (PVOD/PCH) involvement
1.6 Persistent PH of the newborn
GROUP 2 PH associated with left heart disease***
2.1 Heart failure:
2.1.1 with preserved ejection fraction
2.1.2 with reduced or mildly reduced ejection fraction
2.2 Valvular heart disease
2.3 Congenital/acquired cardiovascular conditions leading to post‐capillary PH
GROUP 3 PH associated with lung diseases and/or hypoxia***
3.1 Obstructive lung disease or emphysema
3.2 Restrictive lung disease
3.3 Lung disease with mixed restrictive/obstructive pattern
3.4 Hypoventilation syndromes
3.5 Hypoxia without lung disease (e.g., high altitude)
3.6 Developmental lung disorders
GROUP 4 PH associated with pulmonary artery obstructions**
4.1 Chronic thrombo‐embolic PH
4.2 Other pulmonary artery obstructions
GROUP 5 PH with unclear and/or multifactorial mechanisms (to be expanded by specific pediatric conditions)*
5.1 Hematological disorders
5.2 Systemic disorders
5.3 Metabolic disorders
5.4 Chronic renal failure with or without hemodialysis
5.5 Pulmonary tumor thrombotic microangiopathy
5.6 Fibrosing mediastinitis

*Note:* Asterisks denote the relative frequency of disease subgroups, with * representing low, ** moderate, and *** high frequency.

Globally, PH is recognized as a major health concern affecting people across all age ranges (Humbert et al. 2022). Current estimates suggest that around 1% of the world's population is affected, with prevalence rising significantly in individuals over 65 years, largely due to the increased incidence of cardiac and pulmonary conditions initiating PH in this age group (Hoeper et al. 2016). Left heart disease and chronic lung conditions represent the most frequent causes of PH worldwide (Vahanian et al. 2022). Irrespective of etiology, the development of PH is consistently associated with worsening symptoms, progressive functional decline, increased challenge to the right heart, and an increased risk of mortality (Hoeper et al. 2016).

Research into PH and the right ventricular response to increased afterload is critical to improving early detection, understanding disease mechanisms, optimizing treatment strategies, and ultimately enhancing patient outcomes. However, the heterogeneity of PH and the rarity of some of the PH (sub)‐groups create challenges in accumulating large‐scale, high‐quality data (Galie et al. 2016). National and regional PH registries have become important resources to address this gap, enabling the systematic collection and analysis of real‐world clinical, demographic, and treatment data. These single registries have advanced understanding of regional disease patterns, treatment choice and responses, and outcomes, and have provided important information extrapolated to a global view of PH (Farber et al. 2015; Gall et al. 2017; Swift et al. 2012).

Yet, despite their undisputed value, individual registries are inherently constrained by geographic, demographic, and practice‐specific limitations. To overcome these challenges, the development of a global PH registry that harmonizes and integrates existing national and regional databases offers a transformative opportunity to generate deeper and more generalizable insights. By combining diverse patient populations and healthcare systems and markedly scaling up the number of enrollees, such a registry can enhance the statistical power of research, reveal international variations in care and outcomes, provide evidence for the impact of different altitudes, air pollution conditions, drug accessibility and ethnicities, identify and more deeply characterize rare subtypes, and contribute to the development of universally applicable clinical guidelines.

An international, overarching registry also allows for the integration of heart function metrics and lung‐specific variables to bridge the understanding of cardio‐pulmonary physiology and pathophysiology. This integrative approach enables the exploration of mechanistic links between pulmonary vascular changes and cardiac remodeling. In doing so, it supports the study of disease trajectories, the evaluation of known and the identification of novel cardiopulmonary biomarkers, and the advancement of in‐depth response to therapy profiling, considering both the impact on the lung vascular and the cardiac systems. This approach supports treatment strategies informed by underlying pathophysiology and strengthens efforts to connect genetic markers with clinical disease expression.

To follow this view and to harness large‐scale data, *PVRI GoDeep* was established as a global PH meta‐registry, integrating information from existing local PH registries throughout the world (Majeed et al. 2022). Unlike other international efforts such as PRECISION ALS (McFarlane et al. 2023) or COMPERA (Comparative, Prospective Registry of Newly Initiated Therapies for Pulmonary Hypertension) (Pittrow et al. 2009), which require centers to manually re‐enter their data into a centralized system, thereby duplicating efforts, GoDeep offers a more streamlined solution. Participating centers can retain their current systems while contributing to a unified dataset without manual and duplicate data entry. Our data management team handles the technical work of identifying, mapping, and standardizing the data, minimizing the work on participating sites. Although real‐world data are rarely perfect, it is ensured through close collaboration with each center and a robust quality assurance process that the data integrated into GoDeep meet the highest standards of accuracy and consistency.

This paper outlines the registry's structure, details its robust data framework with a focus on data quality assurance, and highlights the analytical approaches and key outputs, including phenotyping, predictive modeling, and novel research findings.

## 2 Overview

PVRI GoDeep is a global meta‐registry established in 2020 under the umbrella of the Pulmonary Vascular Research Institute (PVRI) and the Justus Liebig University in Giessen, Germany (NCT05329714). Its central data repository structure has been approved by the Ethics Committee of the University of Giessen/University Hospital (AZ 30/19). It is coordinated as a scientific initiative by the Institute for Lung Health (ILH) in Giessen, a member of the German Center for Lung Research (DZL). GoDeep was created to address the growing need for large‐scale, harmonized, real‐world data on PH and right ventricular adaptation versus failure across diverse geographic regions and healthcare contexts. It integrates anonymized, patient‐level data from existing PH registries at expert centers worldwide. Participating centers contribute their data following institutional ethical approval and the signing of a participation agreement.

**FIGURE 1:**
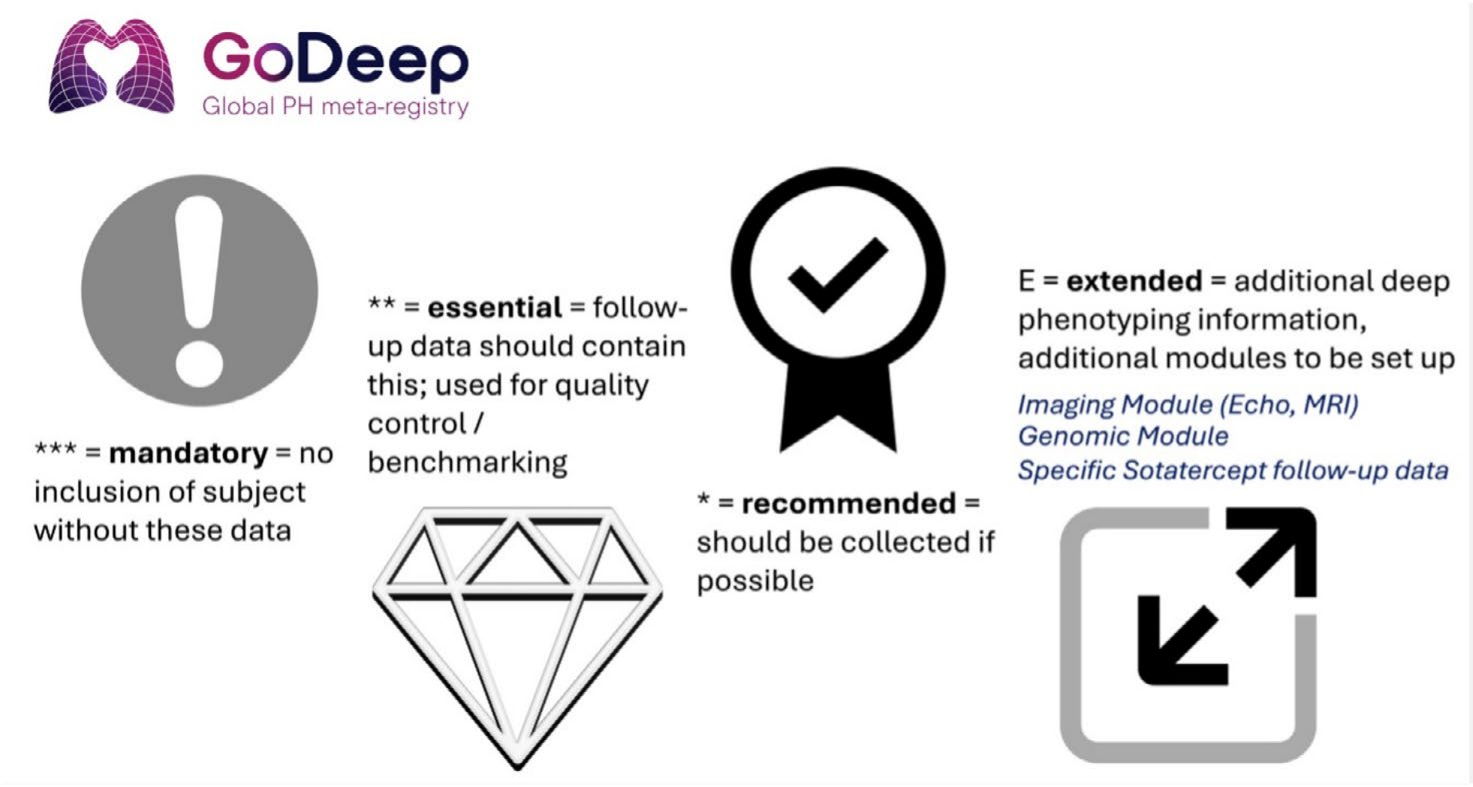
GoDeep Categories of Variable Importance. At the initiation of GoDeep data acquisition, consortium members defined four categories of variable importance: Mandatory variables (e.g., age at diagnosis, sex, right heart catheterization), Essential variables (e.g., WHO functional class, BNP, 6‐min walk distance), Recommended variables, and Extended variables (e.g., imaging, echocardiography, MRI). A complete and up‐to‐date list of all variables is available on the GoDeep webpage: https://ilh‐giessen.de/en/pvri‐godeep/.

Recognizing that individual registries vary in clinical focus and data collection practices, GoDeep initiated a series of consensus meetings, described in more detail in Majeed et al. (2022), to define a dataset with variables categorized as mandatory, essential, recommended, or extended, based on clinical relevance and availability (Figure 1). Mandatory variables are required for patient inclusion in the registry. Without those variables, such as the date of diagnosis, baseline right heart catheterization (RHC) data, and survival status, a patient record cannot be integrated into the dataset. Essential variables are expected to be available at baseline and during follow‐up and play a key role in quality control, longitudinal tracking, and benchmarking. Recommended variables should be collected when possible; although not strictly required, they enhance the depth and analytical value of the data. Extended variables comprise additional deep phenotyping elements, such as CPET (cardiopulmonary exercise testing), imaging, or other specialized data modules, and are collected in selected research contexts to broaden the registry's capabilities over time. This list evolves continuously to incorporate emerging clinical developments, for example new therapies such as sotatercept, and genomic data. All variables are catalogued in a comprehensive data dictionary, which includes both short and long descriptive labels, standardized codes (e.g., LOINC [Logical Observation Identifiers Names and Codes]; (McDonald et al. 2003) and SNOMED CT [Systematized Nomenclature of Medicine—Clinical Terms]; (El‐Sappagh et al. 2018)), agreed‐upon units of measurement, and ranges of physiologically viable values.

As described in Majeed et al. (2022), GoDeep operates under a governed, tiered‐access model in which centers provide data in exchange for full access to the meta‐registry and representation on the steering board, which oversees the scientific use of the data. Public access includes detailed metadata and aggregated summaries. Registered users may perform real‐time feasibility queries on selected data, primarily for consortium members but also for external academic or industry partners, upon case‐by‐case approval by the steering board, with contributing centers retaining autonomy over the inclusion of their data. All subsequent analyses are conducted by a member of the steering board committee, and the underlying data are not made available to third parties.

**FIGURE 2:**
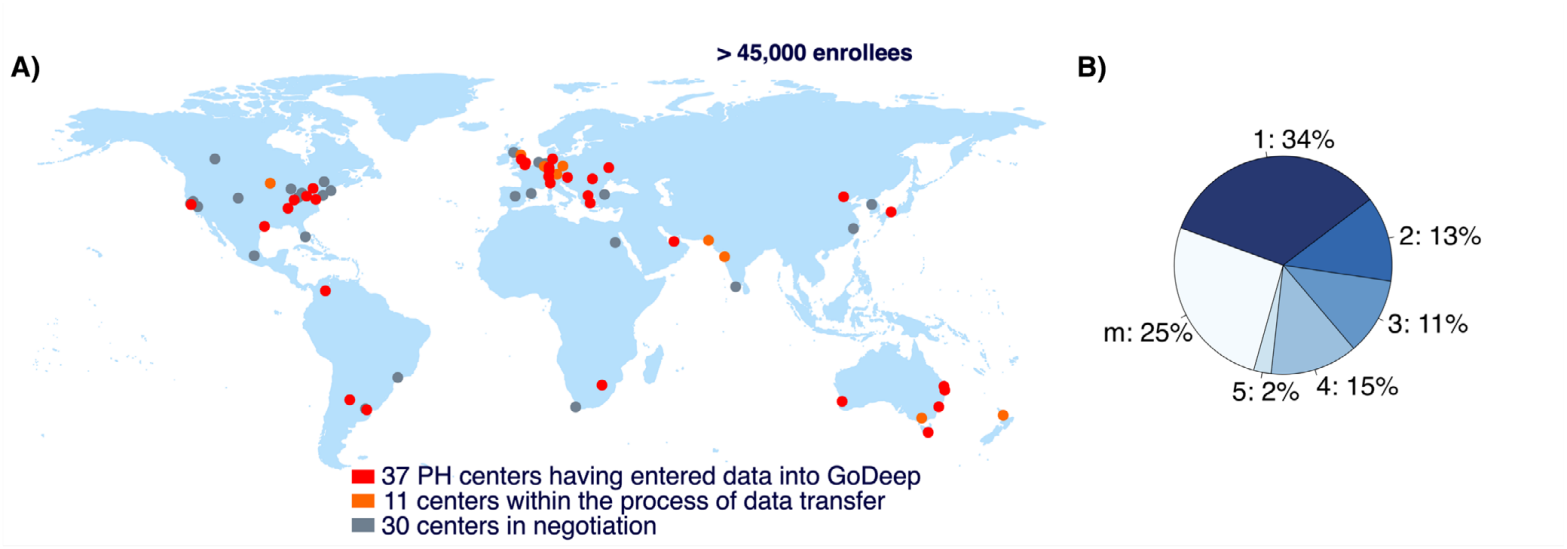
Centers Contributing to GoDeep. (A) World map shows the contributing centers. (B) The pie chart shows the distribution of pulmonary hypertension groups (1–5), as well as patients assigned to multiple groups or not assigned to any specific group (m).

As of January 2026, GoDeep has aggregated data from over 37 centers and encompasses > 45,000 enrolled individuals, with > 39,000 patients with hemodynamically proven PH based on the current definition of mean pulmonary artery pressure > 20 mmHg. A further group includes patients in whom PH was suspected based on clinical and non‐invasive diagnostic criteria, but the RHC undertaken due to this clinical reasoning showed a mPAP ≤ 20 mmHg. An additional 11 centers have signed participation agreements and are actively in preparation for integrating their data, further expanding this diverse resource. The patient cohort spans all continents, with roughly 48% from the United States, 44% from Europe, and 8% from the rest of the world; the latter percentage currently growing with the inclusion of several newly recruited centers (Figure 2).

Patients in the registry were diagnosed with PH between 1980 and 2025, covering the full spectrum of clinical classifications. Within this population, 34% are classified as Group 1, 12% as Group 2, 11% as Group 3, 13% as Group 4, and 3% as Group 5. Additionally, 27% have been assigned to more than one group or remained unclassified (Figure 2). As the PVRI GoDeep meta‐registry exclusively includes patients treated at specialized PH expert centers, the distribution of PH groups does not reflect the global epidemiology of PH. In particular, PAH is over‐represented, consistent with the historical focus of expert centers on PAH and similar observations in other large PH registries. Furthermore, while PAH has traditionally been considered a disease of younger women, contemporary registry data demonstrate a shift toward older and more comorbid patients with a less pronounced female predominance. Accordingly, the age and sex distribution observed in GoDeep are in line with recent real‐world P(A)H cohorts (Gall et al. 2017; Hoeper et al. 2022; Hurdman et al. 2012).

The median follow‐up duration stands at 2.26 years [0.42, 5.5] post‐diagnosis. Overall, 34% of the cohort have deceased. The median age at diagnosis is 63 years [50, 72], and 58% of patients are female. Race data are available for 69% of patients, among whom 81% are White, 12% Black, 5% Asian, and the race of 2% was classified as “other or mixed”, according to internationally accepted definitions (McKenney and Bennett 1994). As to WHO functional class at the time of diagnosis, 3% were classified as Class I, 21% as Class II, 64% as Class III, and 12% as Class IV, reflecting the broad range of disease severity represented in the registry.

## 3 Data Entry Cycle

**FIGURE 3:**
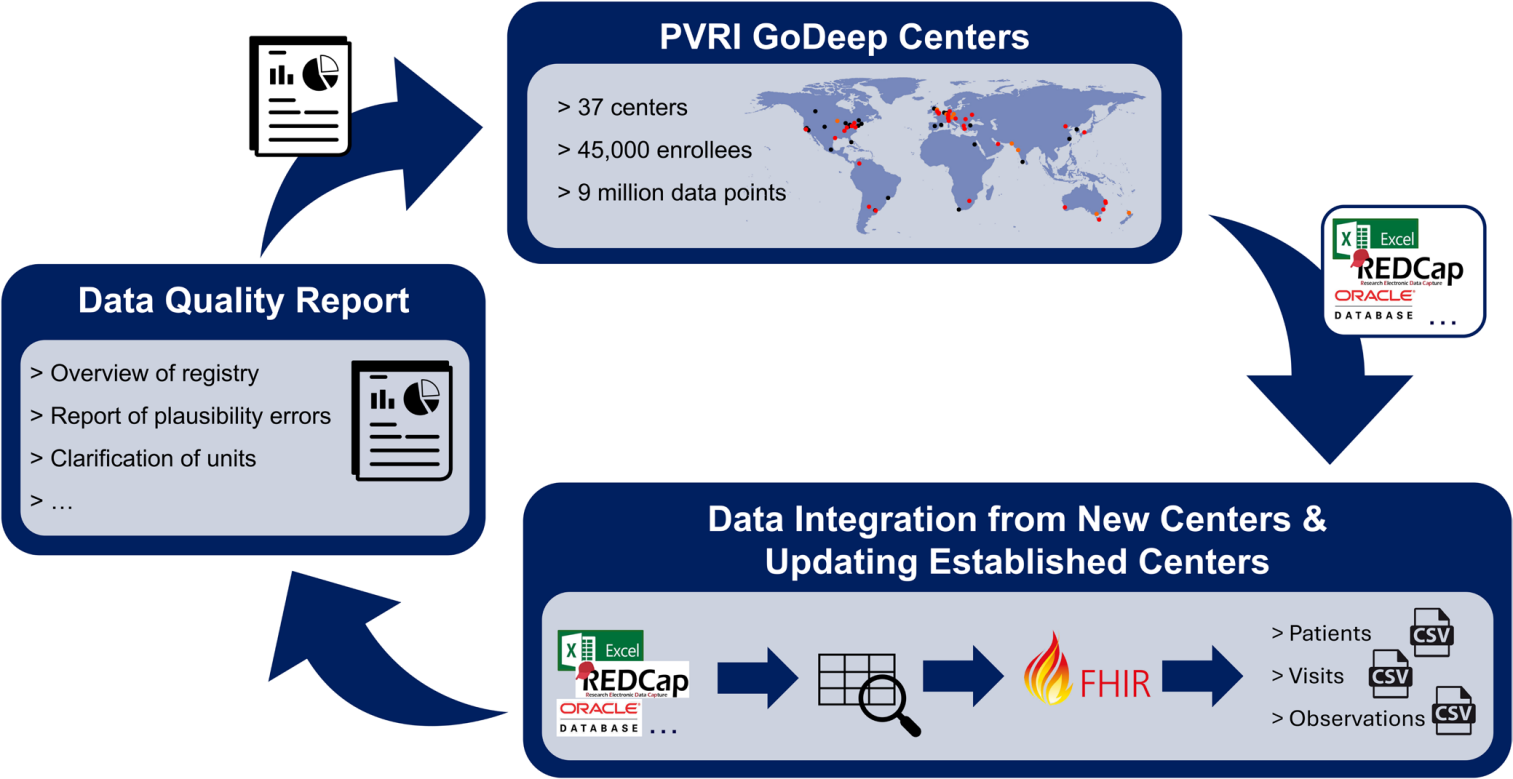
Data entry cycle.

Anonymization procedures follow local regulatory requirements but always involve the removal of identifiable information such as names, addresses, social security numbers, passport or ID numbers, phone numbers, and similar personal data. In addition, dates are modified to further protect patient privacy: This includes anonymizing birth and diagnosis dates, either by generalizing them to the decade or by shifting them within a predefined narrow time window. As the date of diagnosis, the first right heart catheterization fulfilling the hemodynamic definition of PH (Kovacs et al. 2024) is used. All other dates are expressed relative to the diagnosis date, typically in months before or after diagnosis. Anonymization is performed locally before transferring the data to GoDeep, with an anonymization script provided by GoDeep central if needed (e.g., K‐anonymization). All further transfers, processing and analyses is done on this anonymized data set. After transfer to GoDeep, each submitted dataset is first assessed for structural compatibility and completeness, with a particular focus on the availability of mandatory variables and dates. Once the mandatory variables have been identified and their completeness confirmed, our data management team uses the data dictionary to map all other variables to the corresponding standardized codes. This harmonization is essential, as centers often use different abbreviations, descriptive labels, and, in non‐English‐speaking countries, their native languages. After the mapping is complete, the data are converted into three standardized CSV files: *patients*, *encounters*, and *observations* using FHIR (Fast Healthcare Interoperability Resources) format. The *patients* file contains key demographic information: a GoDeep patient ID (assigned by us), the original center‐specific ID created after anonymization, gender, and dates of birth and death (if applicable), both expressed relative to the anonymized diagnosis date, as well as vital status. The *encounters* file lists all dates on which data points were collected upon visits, with an encounter ID for each generated by us. The *observations* file contains individual data points, each linked to the GoDeep patient ID and corresponding encounter ID, along with the standardized codes, start dates, data type (e.g., numeric), value, and unit, each specific to the respective observation. The end dates are collected when applicable/available (Figure 3).

It is important to note that patients can be mapped to different PH classifications based on the classification the center is using. Furthermore, because detailed right heart catheterization data are available, post hoc reclassification is possible. This approach has been demonstrated in a recent publication on the treatment of patients with mild PH (Yogeswaran, Funderich, et al. 2025), who met the diagnostic criteria according to the 2022 ESC/ERS guidelines but would not have been classified as having PH under the 2015 guidelines (Galie et al. 2015; Humbert et al. 2022).

The dataset is then enriched with derived and aggregated variables. These include survival time (in months) from diagnosis, the number of distinct PH diagnoses, assignment to PH diagnosis (groups, subgroups, overlapping groups), the number of recorded visits, PAH specific medication (broken down by drug classes), oxygen therapy, and geographic details such as the city, country, and continent of the PH expert center for each patient. Additionally, comorbidities are mapped to standardized disease groups, and relevant classifications are derived from numeric variables, for example, ascribing to underweight, normal weight, overweight, and obesity based on body mass index (BMI) according to WHO/CDC standards (CDC.gov 2024; de Onis et al. 2007). The original, more granular data remain preserved and can be accessed for analysis if needed.

Data quality checks are carried out as part of the quality assurance process (detailed in the next section). The results are compiled into a data quality report to be provided to the individual centers, along with the number of enrolees, the units identified for each variable, and an overview of data availability. The data availability is presented in two ways: (i) a table by data domain (e.g., laboratory, RHC, echocardiography), showing the number of enrolees with data for each variable, the corresponding percentage of the total cohort, and the average number of records per patient; and (ii) a bar plot grouped by classification (mandatory, essential, recommended and extended) to visualize overall coverage. This report is sent to the respective center along with any outstanding queries. The medical and data management team at each center reviews the report and provides feedback concerning missingness, discrepancies, and any further issues. If necessary, they resubmit a corrected version of their data. Should further clarification be required, a video meeting is arranged between their team and GoDeep central.

As soon as all queries have been resolved, the data is included into the central meta‐registry and used for analyses. The center then enters a regular update cycle, typically every 3–4 months. During each cycle, the center provides an updated data export file, which is again reviewed as described above, with putative adjustments concerning interim changes (e.g., changes in the local data storage systems or export configurations). Each new export then fully replaces the previous one in the meta‐registry, thus allowing capturing of long‐term data. Otherwise, already reported data could not be merged with previous patient specific records due to the anonymization process. A new data quality report, including any additional queries, is then again sent to the center for clarification and feedback.

## 4 Data Quality Assurance

While this update cycle is continuously ongoing for both new and existing centers, a snapshot of the current state of the registry for extraction of a fixed dataset for analytical purposes can be made at any time. Prior to capturing this snapshot, rigorous data quality checks are again carried out. Any errors identified during these checks are compiled in an additional data quality report and shared with the centers for review and correction. As long as there is no feedback for correction, data identified as putatively incorrect are excluded from the analytical procedure. The quality assurance processes generally focus on five key dimensions: timeliness, validity, consistency, completeness, and accuracy.

### 4.1 Completeness

A data completeness check is essential to ensure that all required information is present and preserved throughout the ETL (extract, transform, load) process. During data transformation, even minor formatting inconsistencies, such as incorrect decimal separators, spelling mistakes, and inconsistent use of labels and date formats, can lead to considerable amounts of data being unusable because they cannot be transferred. The feedback report summarizes the availability of each variable across all patients to be compared to the original dataset submitted by the center. It is important to note that variables flagged as putatively incorrect during validation are excluded from the availability summaries and reported separately. As such, data availability reflects only the usable portion of the data and not the full submission.

### 4.2 Timeliness

A reasonable refresh cycle is essential to capture ongoing clinical developments, support longitudinal analyses, and enhance the overall utility of the registry for research purposes. To this end, and to ensure continued relevance, regular data updates are scheduled every 3 months.

### 4.3 Validity

As part of the data validation process, several checks are performed to ensure plausibility as well as internal and external consistency. An important tool in this process is the use of boxplots to visually assess the distribution of each variable within the reporting history of an individual center, as well as across centers, allowing for the quick identification of “outlying submissions”, which might indicate incorrectly used units for the respective variable or systemic deviance of data assessment procedures as compared to all other centers.

### 4.4 Consistency

Ensuring consistency with the original records remains the responsibility of the contributing centers. However, when multiple entries exist for values that should remain constant, such as birth date, diagnosis date, diagnosis‐defining right heart catheter‐derived variables, WHO functional class at study entry, or date of death, these discrepancies are identified and reported back to the respective centers for clarification or correction. Until such issues are resolved, the affected patients are flagged and excluded from any further analyses.

A particular challenge arises when patients transfer between hospitals. To avoid potential duplicate entries, cross‐center checks are conducted using key variables such as detailed diagnosis‐defining right heart catheterization data, age at diagnosis, birth decade, and diagnosis decade. Applying these GoDeep‐based “identifiers,” less than 1 per mille of data indicated putatively across‐center duplicates, which could be clarified by feedback from the respective centers in each case. This procedure ensures that each patient is represented only once in the GoDeep dataset.

### 4.5 Accuracy

As part of the accuracy dimension within the quality assurance process, both plausibility and coherence checks are conducted. During the plausibility check, values that are incompatible with life are identified and excluded, using thresholds adapted to the expected range for patients with PH. These thresholds are defined by clinical experts and maintained within our data dictionary, with updates as needed. Values outside the range of physiological or pathophysiological viability are flagged and not entered into any review process until clarification by feedback from the respective PH referral center.

Coherence checks involve assessing the internal consistency of physiologically and/or methodologically related variables, such as the interdependence between systolic, diastolic, and mean pulmonary arterial pressure (mPAP), pulmonary arterial wedge pressure (PAWP), cardiac output (CO), and pulmonary vascular resistance (PVR), or the interdependence of oxygenation, oxygen content, cardiac output, and central venous oxygen saturation. If a set of values is found to be insufficiently coherent, it is flagged and reviewed by our medical advisory team. Unless the clinicians indicate that one or more values in the set are incorrect, all values are retained and used in the analysis.

## 5 Preparing Data for Analysis

Once all necessary modifications have been completed, the data extraction and preprocessing steps begin, initiating the process by which clinical research questions are translated into structured specifications. The research process begins with a clinical question formulated by medical experts. Based on this research idea, they define the subset of patients relevant to the analysis, the baseline and follow‐up periods (if applicable), and the specific variables required. These specifications are then translated into a structured project configuration file, which outlines the cohort definition and variable selection in a standardized yet human‐readable format. A custom R script then parses the project configuration file and combines it with internal logic to extract the relevant patient‐level data. The result is a tabular text file (comma separated values, CSV) following the structure “cases in rows, variables in columns”, in which each row contains data for a single patient.

The extracted values of time‐varying variables such as laboratory values, RHC, measurements, or echocardiographic data, are those closest to the desired time‐point (as given in months after diagnosis) that exist within a predefined (time) window around the given time point. If there is no recorded value for the patient within this time window, the value is “missing”. If several desired time‐points are given, e.g., to analyse changes over time, this process is repeated for each given time‐point, resulting in several columns in the CSV file. For each of these columns, two additional columns are added, indicating the time spans from the measurement to the given reference time point and to the time of diagnosis, respectively.

Creating a structured project configuration file that thoroughly documents the selection decision ensures traceability and allows the dataset to be consistently reproduced at a later time. Additionally, by focusing only on the data relevant to the respective analysis, the amount of data is reduced, thereby considerably enhancing clarity and providing a better overview of the dataset for the analyst.

### 5.1 Automatic Data Completion

The data in the CSV file is then automatically completed in two ways: (a) columns for additional derived variables are added, and (b) missing values are completed by calculation from given values of other variables. For instance, data from pulmonary function tests will always be given as absolute values and as percent predicted values. The calculations from absolute values to %predicted values in pulmonary function tests are used as shown in Stanojevic et al. (2017) and Bowerman et al. (2023). Missing BNP values were calculated from available N‐terminal pro‐B.type natriuretic peptide (NT‐proBNP) measurements using the relationship $\ln\left(BNP\right)=\frac{\ln(NT‐proBNP)‐0.79}{1.348}$; conversely, missing NT‐proBNP values were obtained by algebraic rearrangement of this equation (Rorth et al. 2020). If one of these variables is not part of the CSV file, it will be added. Missing values in a variable may be calculated from given values of other variables. The calculations are iteratively repeated, including already calculated values, until no more missing values can be calculated. For example, consider a patient for whom body surface area (BSA), cardiac index (CI), mPAP, and PAWP are given, but CO and PVR are missing. In a first step, CO is calculated as CO = CI*BSA, and in the next iteration, PVR is calculated as PVR = (mPAP‐PAWP)/CO, using the previously calculated CO value. Each computed value is checked for falling outside the range of viability.

### 5.2 Data Enrichment by Imputation

A common characteristic of registry and real‐world data is that values are missing, which cannot be obtained by feedback from the original referral center or derived (re‐calculated) from existing variables, as discussed above. In this case, statistical evaluations will either be performed on the basis of the given “missingness” with the available data set (checking that missingness is distributed at random, thereby avoiding a bias; see discussion below), or missing values are imputed using multiple imputation by chained equations (MICE) with predictive mean matching (pmm) (Van Buuren and Groothuis‐Oudshoorn 2011). Predictive mean matching imputes missing values by finding observed cases with predicted values closest to the missing case's predicted value, then randomly selecting one observed value as the replacement. Typically, all available variables known to be predictive are included in the imputation model, with the specific variables imputed chosen according to the research question. This method generates realistic imputations within the observed data range, works for both continuous and discrete variables, and is relatively robust to model misspecification (Van Buuren and Van Buuren 2012). MICE in general avoids the high data loss of listwise deletion and the distortions and artificial homogeneity of mean imputation.

**FIGURE 4:**
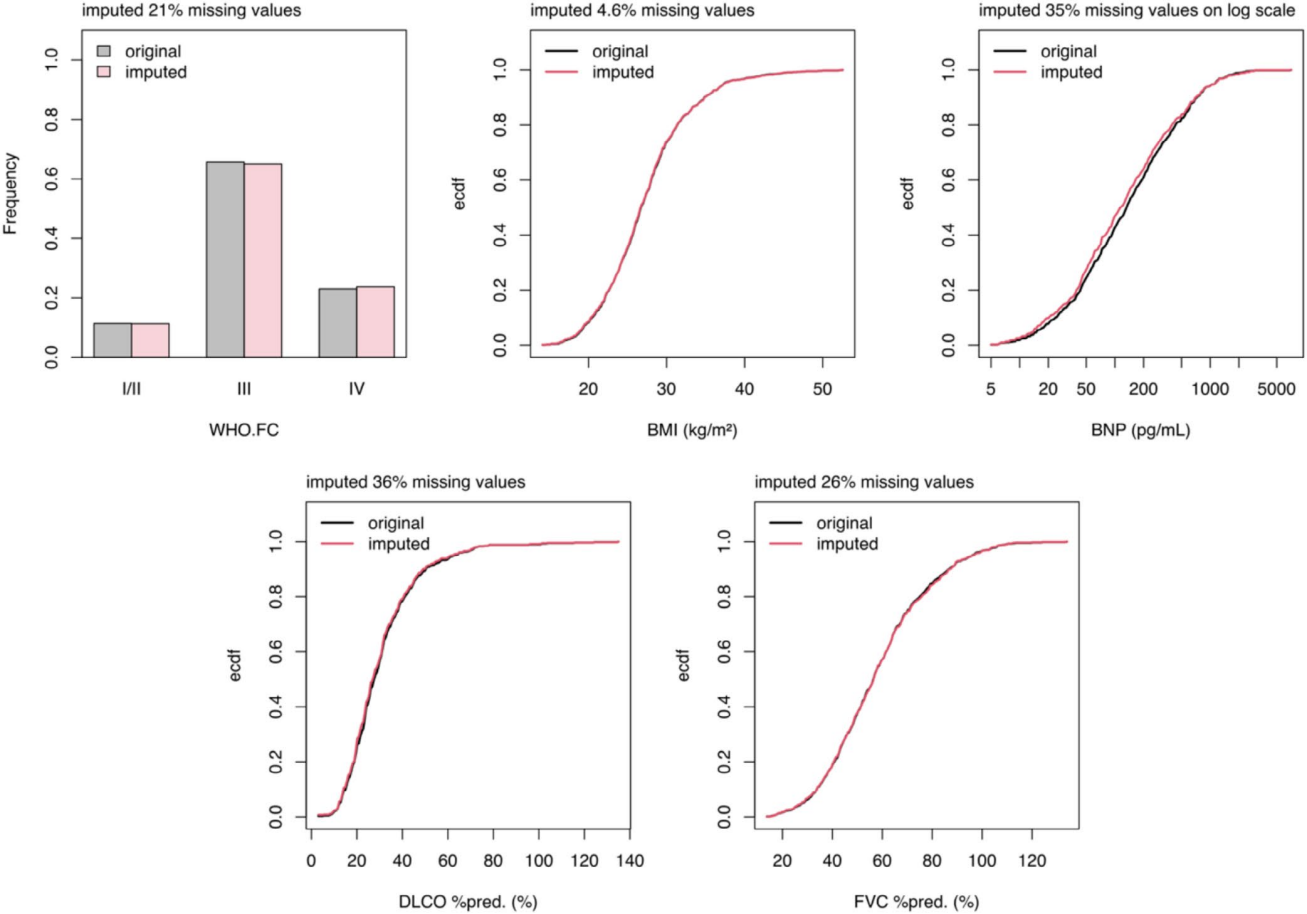
Examples of a comparison of the empirical cumulative distributions before and after imputation from Yogeswaran, Hassoun, et al. (2025). The diagrams present the empirical cumulative distributions of the variables subjected to imputation. The black line represents the distribution of the observed data, while the red lines depict the distributions after incorporating the imputed values.

Since the cumulative baseline hazard is unknown, the Nelson–Aalen estimator is applied to approximate it during the imputation process. Following imputation, a thorough sanity check is conducted to assess the quality and reliability of the imputed values. This involves comparing the empirical cumulative distribution functions of the original observed data against the combined dataset that includes both observed and imputed values. These figures (an example given in Figure 4 (Yogeswaran, Hassoun, et al. 2025)) help verify that the imputed data maintain a realistic distribution consistent with the original data, ensuring the imputation process does not introduce bias or distortions. Imputation is based on the assumption that data are missing completely at random or minimum, missing at random. To examine the plausibility of this assumption, the correlation between missingness, defined as the total number of missing values across all variables for each patient, and the survival outcome is assessed, using the parameter of interest (e.g., phosphodiesterase‐5 inhibitors and survival in the example figure). This missingness indicator is included in the model of interest as a predictor of survival and tested for significance by a likelihood ratio test. To capture potential nonlinear relationships between missing data and the log hazard, the missingness variable is modeled using natural splines with one, two, and three degrees of freedom, allowing for linear, quadratic, and cubic effects. The plausibility of imputed values is assessed by diagnostic plots, for instance comparing the empirical distributions of data with and without imputed values.

## 6 Application Examples

**FIGURE 5:**
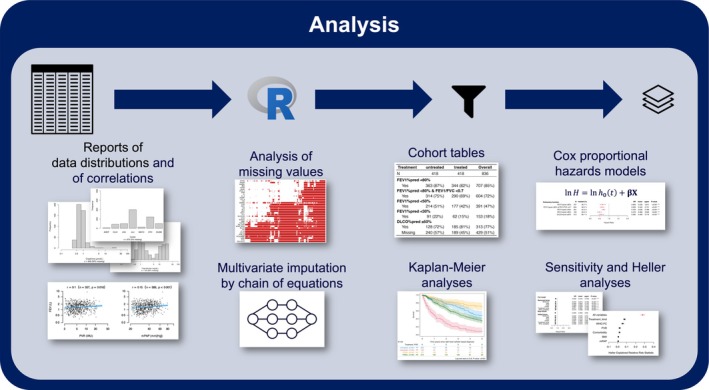
Schematic of standard analysis pipeline.

Harmonized GoDeep data was successfully used in several projects addressing a variety of questions that require data from large cohorts of PH patients (Table 2). These analyses apply a wide range of statistical methods suited to the structure and complexity of the data and the respective research questions. Our approach combines Cox survival models with more flexible modeling tools and a set of sensitivity checks to ensure the reliability of results (Figure 5).

**TABLE 2:** Categories of evaluation.

Category	Example
“Classical” PAH (group 1) focus	Risk Scores (*Chest* 2024, 166(3), 585–603)
Specific PAH subgroups	“Mild PAH” (*ERJ Open Res* 2025, 11(1))
	PoPH (*Pulm Circ* 2025, 15(3), e70121)
Non‐PAH PH	COPD‐PH (*Chest* 2025, 167(1), 224–240)
	PH‐ILD (*AJRCCM* 2025)
The global PH view	Impact of Sex and Race (Yogeswaran, Annis, et al. 2025)
	The systemic arterial hypertension paradox in PH (under review)
	Obesity as survival confounder in PH (under review) Pulmonary Artery Compliance and Prognosis Across the Spectrum of Pulmonary Vascular Diseases (under review)
Enhancement of (global) trial design and in silico studies	

One key application has been the validation of risk stratification models, such as REVEAL 2.0, across all major PH groups (Yogeswaran et al. 2024). This study has shown that tools originally developed for Group 1 PAH retain prognostic value in other PH subgroups, helping to improve patient risk assessment and clinical decision‐making across broader populations. The analysis employed Cox proportional hazard models and discrimination metrics, such as c‐statistics and akaike information criteria values, to evaluate the predictive performance of various risk scores for mortality outcomes. The primary cohort included over 8500 patients with detailed hemodynamic data.

**FIGURE 6:**
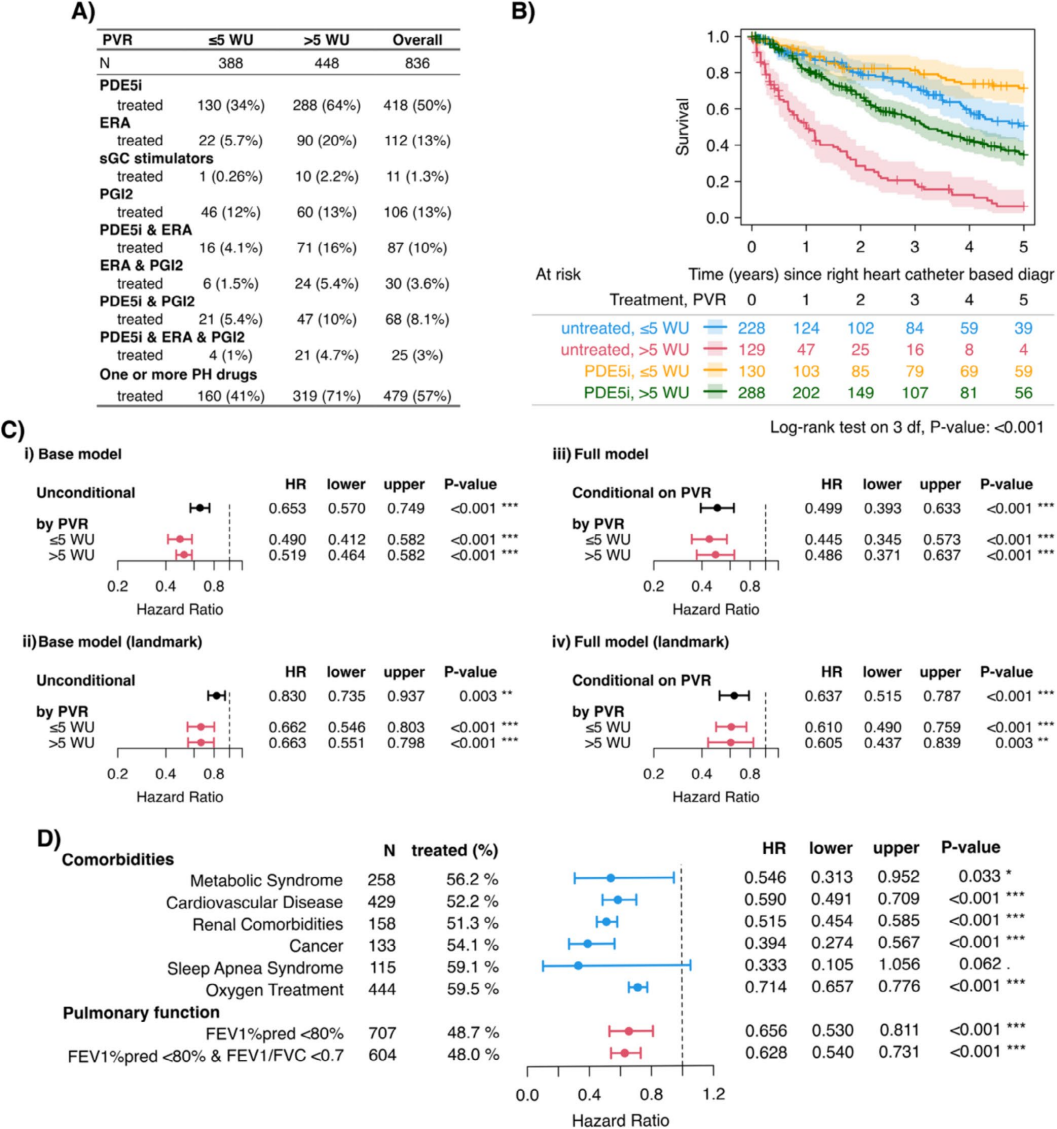
PDE‐5 Inhibitor Therapy in COPD‐PH: Survival Outcomes. Modified from Tello et al. (2025). (A) Use of PH‐targeted therapy among COPD‐PH patients in the GoDeep meta‐registry, stratified by hemodynamic severity. The table reports the number and percentage of patients receiving each type of PH‐targeted therapy within different severity groups. The final row summarizes all patients who received at least one PH medication. (B) Overall survival of COPD‐PH patients, stratified by PDE5i treatment status and hemodynamic severity. Kaplan–Meier curves with 95% confidence intervals are presented for patients treated with PDE5i versus those not receiving any PH‐targeted therapy, further divided into groups with PVR ≤ 5 WU and PVR > 5 WU. (C) Hazard ratios for patients receiving PDE5i treatment compared with those without PDE5i treatment, derived from Cox proportional hazards models. Diagrams display the estimates with 95% confidence intervals. *p*‐values are based on Wald *z*‐tests. (i) Results from the base model. The unconditional estimate represents the HR of PDE5i, adjusted only for age. Estimates for the separate PVR groups are obtained from the base model including the interaction term between dichotomized PVR and PDE5i treatment. This model uses non‐imputed data only. (ii) Results from the base model, as in (i), but after applying the landmark approach to reduce immortal time bias. (iii) Results from the full model. The estimate conditional on PVR is adjusted for a linear relationship between PVR and the log hazard, as specified in the full model. Estimates for the separate PVR groups are obtained in the same way as in (i). This model uses imputed data for variables with missing covariates. (iv) Results from the full model, as in (iii), but after applying the landmark approach. (D) Association of PDE5i treatment with comorbidities and pulmonary function impairment. Results are based on the base model applied to subgroups defined by comorbidities and pulmonary function. Hazard ratios compare patients receiving PDE5i with those not treated, derived from Cox proportional hazards models. Diagrams display estimates with 95% confidence intervals, and *p*‐values are based on Wald *z*‐tests. COPD, chronic obstructive pulmonary disease; ERA, endothelin receptor antagonists; FEV1% pred, forced expiratory volume percent predicted; FVC, forced vital capacity; HR, hazard ratio; lower and upper, lower and upper limits of the 95% confidence interval of the HR; PDE5i, phosphodiesterase‐5 inhibitors; PGI2, prostaglandin I2 and its analogues (inhalative or parenteral); PH, pulmonary hypertension; PVR, pulmonary vascular resistance; sGC, soluble guanylate cyclase; WU, wood units.

GoDeep has also supported analyses of the use and impact of therapies beyond their approved indications for pulmonary hypertension. For instance, phosphodiesterase‐5 inhibitors (PDE5i), while primarily recommended for patients with pulmonary arterial hypertension (PAH; group 1), are broadly used off‐label in other PH groups. One such group includes patients with PH due to chronic obstructive pulmonary disease (COPD‐PH). To examine whether PDE5i therapy is associated with improved survival in this population, data from the registry were extracted and analysed (Tello et al. 2025). Although COPD‐PH patients are typically excluded from randomized trials investigating PAH‐specific drugs, our findings suggest a potential survival benefit in those receiving PDE5i treatment, as shown in summary Figure 6. In another study, GoDeep data were used to investigate the use and outcomes of targeted therapies in portopulmonary hypertension, offering new insights into treatment patterns in this rare PH subtype (Jose et al. 2025).

A notable example of GoDeep's added value is the analysis of patients with so‐called “mild” pulmonary hypertension, as presented in Yogeswaran, Funderich, et al. (2025). In 2018, the clinical definition of PH was revised by the 6th World Symposium on Pulmonary Hypertension, lowering the diagnostic threshold for mPAP from ≥ 25 mmHg to > 20 mmHg. As a result, patients with mPAP between 20 and 25 mmHg, previously not classified as having PH, were newly recognized as part of the disease spectrum. These patients were, however, excluded in all pivotal trials underlying market access of PAH‐specific drugs, and they remain a small minority in most cohorts, making robust analysis at the level of individual centers difficult. By pooling data across multiple international sites within GoDeep, this limitation is overcome, enabling a comprehensive analysis of this understudied subgroup and providing first evidence for the efficacy of PAH‐specific medication also in the sub‐cohort with “mild PH”.

In the most recent publication, Hemodynamics and PDE5i Treatment Associated with Survival in Pulmonary Hypertension in Interstitial Lung Disease (Yogeswaran, Hassoun, et al. 2025), a significant association was observed between PDE5i use and survival in PH patients with PVR > 5 wood units (WU), but not for patients with PVR below 5 WU. Another example examined sex‐based differences in survival among more than 21,000 patients with pulmonary hypertension across different disease conditions (Yogeswaran, Annis, et al. 2025). Male patients consistently showed higher mortality than females for patients with different ages, disease severities, comorbidities, and treatments.

In all these application examples, it was important to rigorously evaluate the proportional hazards assumption. To this end, diagnostic plots of Schoenfeld residuals are routinely generated, while deviance residuals are examined to uncover influential data points that could destabilize the model. Beyond assumption testing, model performance is explored using Heller's Explained Relative Risk, which highlights the proportion of total explained risk attributable to individual covariates. This not only quantifies predictive accuracy but also provides a clear view of how strongly each variable impacts survival outcomes. Robustness is assessed by systematically modifying Cox proportional hazards models, sequentially adding or removing individual covariates and groups of covariates. Such iterative refinement allows a deeper understanding of the stability of effect estimates and the sensitivity of findings to specific modeling choices, thereby strengthening transparency and interpretability. In selected cases, analyses contrast a streamlined base model, containing only the core covariates necessary for adjustment (e.g., age at diagnosis, diagnosis decade, center of origin, sex and treatment information for PDE5i), with an expanded full model that incorporates additional variables (e.g., WHO functional class, body mass index, PVR, mPAP, Brain natriuretic peptide, forced vital capacity percent predicted and diffusing capacity of the lung for carbon monoxide percent predicted), as applied e.g., in Yogeswaran, Hassoun, et al. (2025). Comparing these two models highlights the added value of additional covariates, revealing not only their statistical contribution but also their potential clinical significance.

For survival analyses investigating treatment effects, landmark analysis is applied to reduce immortal time bias, the distortion that occurs when patients must survive a predefined period before becoming eligible for treatment, particularly when therapy is not initiated immediately after diagnosis. In Tello et al. (2025), for instance, the landmark was set at 3 months following the initial RHC. Only patients who survived at least this 3‐month period and were either untreated with PDE5i or initiated therapy within that timeframe are included in the analysis. Survival times are then recalculated by subtracting the 3‐month landmark. An alternative approach to minimize immortal time bias is the target trial emulation framework, which seeks to replicate a randomized controlled trial (RCT) by explicitly defining eligibility criteria, baseline, treatment strategies, and follow‐up in order to estimate causal effects, with the aim of replicating a hypothetical trial using existing data (Hernan et al. 2022).

To assess the robustness of our estimated treatment effect to potential unmeasured confounding, a dual‐parameter sensitivity analysis is carried out using the survSensitivity method based on the R package survSens (Huang et al. 2020; Huang 2023). This approach evaluates how the estimated log hazard ratio for the treatment varies with hypothetical levels of unmeasured confounding, represented by sensitivity parameters on both the log hazard ratio scale and the probit scale (ζ_Z_). Contour plots illustrate the range of sensitivity parameters for which the intervention effect (e.g., treatment of PAH‐specific drugs) remains statistically significant, providing a rigorous evaluation of the stability of the findings under varying assumptions about unmeasured confounding.

These use cases highlight the potential of GoDeep to support analyses across a broad range of patient populations, using harmonized real‐world data from multiple international centers. By reflecting real‐world clinical practice, GoDeep helps address research questions that may be difficult to study through prospective trials, offering additional insights that can generate interesting research hypotheses and inform care in PH and right heart adaptation.

## 7 Challenges and Perspective

Working with data from diverse sources presents inherent challenges. International collaborations must comply with country‐specific privacy laws, ethics approvals, and data‐sharing agreements, which can complicate or delay data integration. In addition, differences in clinical practice, documentation standards, and data completeness across sites mean that careful harmonization is required. Terminologies and formats must be aligned and missing or inconsistent values must be carefully handled to preserve analytical validity. These steps, spanning data cleaning, transformation, and validation, are time‐intensive but crucial to ensuring the resulting dataset is both robust and suitable for high‐quality research.

Furthermore, integrating data from international registries requires close coordination between the data management team, medical experts, and participating centers. In cases of medical ambiguity, consultation with clinicians is essential to ensure correct interpretation. Different measurement units must be standardized to ensure strict consistency. A detailed account of these challenges and the solutions implemented is provided in Fuenderich et al. (2024).

Additional challenges arise from the current classification of PH patients into groups 1–5 (Humbert et al. 2022). For example, patients with HFpEF may be misclassified as Group 1, as recent studies suggest (Reddy et al. 2024). Such misclassifications can be addressed using detailed clinical data, RHC measurements and imaging data (ECHO, cardiac MRI), which are available in GoDeep and could inform further refinement of patient categorization. Moreover, GoDeep is exploring the inclusion of RHC exercise data, which would allow assessment of PVR increase, CO increase, and PAWP increase upon exercising, further enhancing phenotyping accuracy. Also, despite the large sample size, certain rare PH subtypes may still have limited numbers, limiting generalizability and the confidence in conclusions and statistical analyses, as we see in Group 5.

With the ongoing expansion of GoDeep, the integration of new therapies, biomarkers, or updated clinical guidelines may necessitate revisions to variable definitions, potentially leading to discrepancies with previously collected data.

Despite these challenges, the GoDeep framework offers substantial advantages. By enabling the re‐use of existing infrastructure and minimizing redundant data entry, it lowers the barrier for participation and enhances scalability. Its modular design supports deep phenotyping and future extensions, such as linkage with genomic and omics data or imaging and emulating target trials. Most importantly, GoDeep creates a research‐ready environment for longitudinal, patient‐level analysis on an international scale, offering new perspectives for precision medicine, outcome benchmarking, and real‐world evidence generation in pulmonary hypertension and right heart adaptation. The principles and infrastructure behind GoDeep are disease‐agnostic, making this framework readily transferable to other medical domains that rely on high‐quality, longitudinal real‐world data.

## Funding

This work is funded by the Pulmonary Vascular Research Institute (PVRI).

## Conflicts of Interest

MFünderich has nothing to disclose. AYogeswaran reports non‐financial support from the University of Giessen during the conduct of the study, research grants from the German Research Foundation, support for attending a meeting from OrphaCare and AOP, and personal fees from MSD and Ferrer outside the submitted work. PJanetzko has nothing to disclose. KTello has received personal fees from Bayer, AstraZeneca, Gossamer. WSeeger has received consultancy fees from United Therapeutics, Tiakis Biotech AG, Liquidia, Pieris Pharmaceuticals, Abivax, Pfitzer, Medspray BV. RWMajeed and JWilhelm have nothing to disclose.

## Supporting information


**Table S1:** Example of equations used for variable conversion and consistency checks. The equations in the table are used for variable conversions and consistency checks, and any necessary rearrangements of these equations are applied. The Du Bois formula is used to calculate body surface area. The BNP to NT‐proBNP conversion is based on data from Rorth et al. (2020), with a total least squares regression model fitted to determine the intercept and slope.

## Data Availability

Data sharing not applicable to this article as no datasets were generated or analysed during the current study.

## GoDeep Consortium

Mauro Acquaro (Division of Cardiology, Fondazione IRCCS Policlinico San Matteo, 27100 Pavia, Italy), Imad Al Ghouleh (Cardiovascular Research Center, Brown University Health Cardiovascular Institute, Department of Medicine, The Warren Alpert Medical School of Brown University), James Anderson (Sunshine Coast University Hospital), Jeffrey S. Annis (Division of Cardiovascular Medicine, Vanderbilt University Medical Center, Nashville, Tennessee, USA), Anastasia Anthi (51st Department of Critical Care and Pulmonology Medicine, National and Kapodistrian University of Athens School of Medicine, ‘Evangelismos’ Hospital, Athens, Greece), Alexandra Arvanitaki (61st Department of Cardiology, Aristotle University of Thessaloniki, Greece), Aparna Balasubramanian (Division of Pulmonary and Critical Care Medicine, Department of Medicine, Johns Hopkins University School of Medicine, Baltimore USA), Felix Ballmann (Clinic III for Internal Medicine (Department of Cardiology), Heart Center at the University Hospital Cologne, Kerpener Str. 62, D‐50937 Cologne, Germany), Harm Jan Bogaard (Amsterdam UMC), Evan Brittain (Division of Cardiovascular Medicine, Vanderbilt University Medical Center, Nashville, Tennessee, USA), Hugh Buzacott (Department of Respiratory Medicine, The Alfred Hospital, Melbourne, VIC, Australia), Hector R. Cajigas (Division of Pulmonary and Critical Care Medicine, Mayo Clinic, Rochester (USA)), John Cannon (Royal Papworth Hospital Cambridge (UK)), Stephen Y. Chan (University of Pittsburgh (USA)), Victoria Damonte (University of Cordoba (Argentina)), Effrosyni Dima (1st Department of Critical Care and Pulmonology Medicine, National and Kapodistrian University of Athens School of Medicine, ‘Evangelismos’ Hospital, Athens, Greece), Philipp Douschan (University Hospital Graz, Graz (AT)), Nathan Dwyer (Royal Hobart Hospital), Diego Echazarreta (Universidad Nacional de La Plata (Argentina)), Christina A. Eichstaedt (Center for Pulmonary Hypertension, Thoraxklinik Heidelberg gGmbH at Heidelberg University Hospital and Translational Lung Research Center Heidelberg (TLRC), German Center for Lung Research (DZL), Heidelberg, Germany; Laboratory for Molecular Genetic Diagnostics, Institute of Human Genetics, Heidelberg University, Heidelberg, Germany), Jean Elwing (University of Cincinnati), Kai Förster (Ludwig‐Maximillians‐University Munich (Germany)), Robert Frantz (Department of Cardiovascular Medicine, Mayo Clinic, Rochester, MN USA), Marlize Frauendorf (Milpark Hospital, Johannesburg (ZA)), Stefano Ghio (Division of Cardiology, Fondazione IRCCS Policlinico San Matteo, 27100 Pavia, Italy), Hossein‐Ardeschir Ghofrani (Department of Internal Medicine, Universities of Giessen and Marburg Lung Center (UGMLC), Member of the German Center for Lung Research (DZL), Giessen (Germany); Institute for Lung Health (ILH), Cardio‐Pulmonary Institute (CPI), Giessen (Germany)), George Giannakoulas (1st Department of Cardiology, Aristotle University of Thessaloniki, Greece), Friedrich Grimminger (Department of Internal Medicine, Universities of Giessen and Marburg Lung Center (UGMLC), Member of the German Center for Lung Research (DZL), Giessen (Germany); Institute for Lung Health (ILH), Cardio‐Pulmonary Institute (CPI), Giessen (Germany)), Ekkehard Grünig (Center for Pulmonary Hypertension, Thoraxklinik Heidelberg gGmbH at Heidelberg University Hospital and Translational Lung Research Center Heidelberg (TLRC), German Center for Lung Research (DZL), Heidelberg, Germany), Lars Harbaum (Division of Respiratory Medicine, Department of Internal Medicine II, University Medical Centre Hamburg‐Eppendorf, Hamburg), Paul M. Hassoun (Division of Pulmonary and Critical Care Medicine, Department of Medicine, Johns Hopkins University School of Medicine, Baltimore (USA)), Melanie Heberling (Division of Pulmonology, Medical Department I, University Hospital Carl Gustav Carus of Technical University Dresden, Dresden, Germany), Anne Hilgendorff (Ludwig‐Maximillians‐University Munich (Germany)), Luke Howard (National Heart and Lung Institute, Imperial College London, London (UK)), Arun Jose (University of Cincinnati), Ernesto Junaeda (University of Cordoba (Argentina)), David G. Kiely (Sheffield Pulmonary Vascular Disease Unit, Royal Hallamshire Hospital, University of Sheffield and National Institute for Health and Care Research Sheffield Biomedical Research Centre (UK)), Ingrid King (Murdoch Children's Research Institute, University of Melbourne, Melbourne, VIC, Australia), Hans Klose (Division of Respiratory Medicine, Department of Internal Medicine II, University Medical Centre Hamburg‐Eppendorf, Hamburg), Ziad Konswa (Division of Pulmonary and Critical Care Medicine, Department of Medicine, Johns Hopkins University School of Medicine, Baltimore (USA)), Gabor Kovacs (University Hospital Graz, Graz (AT)), Philipp Krieb (Department of Internal Medicine, Universities of Giessen and Marburg Lung Center (UGMLC), Member of the German Center for Lung Research (DZL), Giessen (Germany); Institute for Lung Health (ILH), Cardio‐Pulmonary Institute (CPI), Giessen (Germany)), Keiichiro Kuronuma (Pulmonary Hypertension Center, Department of Cardiology, NHO Okayama Medical Center, Okayama (Japan)), Edmund Lau (Royal Prince Alfred Hospital), Melanie Lavender (Fiona Stanley Hospital), Allan Lawrie (National Heart and Lung Institute, Imperial College London, London (UK)), Mona Lichtblau (Division of Pulmonology, University Hospital Zurich), Kurt Marquardt (Department of Internal Medicine, Universities of Giessen and Marburg Lung Center (UGMLC), Member of the German Center for Lung Research (DZL), Giessen (Germany)), Hiromi Matsubara (Pulmonary Hypertension Center, Department of Cardiology, NHO Okayama Medical Center, Okayama (Japan)), Farhan Mubashir (Department of Internal Medicine, Universities of Giessen and Marburg Lung Center (UGMLC), Member of the German Center for Lung Research (DZL), Giessen (Germany); Institute for Lung Health (ILH), Cardio‐Pulmonary Institute (CPI), Giessen (Germany)), Horst Olschewski (Charité‐Universitätsmedizin Berlin, corporate member of Freie Universität Berlin and Humboldt‐Universität zu Berlin, Department of Infectious Diseases and Respiratory Medicine, Berlin, Germany), Mauricio Orozco‐Levi (Fundacion Cardiovascular de Colombia; Clinica Cardiovid; Clinica Neumologica del Pacifico), Karen Osborn (Pulmonary Vascular Research Institute (PVRI) Canterbury (UK)), Joanna Pepke‐Zaba (Royal Papworth Hospital Cambridge (UK)), Alba Ramirez‐Sarmiento (Fundacion Cardiovascular de Colombia), Stephan Rosenkranz (Clinic III for Internal Medicine (Department of Cardiology), Heart Center at the University Hospital Cologne, Kerpener Str. 62, D‐50937 Cologne, Germany; Cologne Cardiovascular Research Center (CCRC), Hospital Cologne and Medical Faculty, Heart Center at the University, University of Cologne, Cologne, Germany), Hani Sabbour (Cleveland Clinic Abu Dhabi (United Arab Emirates)), Sandeep Sahay (Houston Methodist Hospital, Houston (USA)), Khaled Saleh (Cleveland Clinic Abu Dhabi (United Arab Emirates)), Laura Scelsi (Division of Cardiology, Fondazione IRCCS Policlinico San Matteo, 27100 Pavia, Italy), Yuriy Sirenko (P.Shupik National University of the Healthcare of Ukraine, Kyiv (Ukraine)), Siva Sivakumaran (Gold Coast University Hospital), Andrew J. Sweatt (Division of Pulmonary, Allergy, and Critical Care, and Vera Moulton Wall Center for Pulmonary Vascular Disease, Stanford University (USA)), Thenappan Thenappan (Department of Medicine, University of Minnesota, Minneapolis, MN, USA), Ioan Tilea (George Emil Palade University of Medicine (Romania)), Olena Torbas (Strazhensku Cardiology Institute Kiev (Ukraine)), Silvia Ulrich (Division of Pulmonology, University Hospital Zurich), Andrea Varga (George Emil Palade University of Medicine (Romania)), Helen M. Whitford (The Royal Children's Hospital; Faculty of Medicine, Nursing and Health Sciences, Central Clinical School, Monash University, Melbourne, VIC, Australia), Christoph B. Wiedenroth (Kerckhoff Klinik and German Centre for Lung Research (DZL/UGMLC)), Martin R. Wilkins (National Heart and Lung Institute, Imperial College London, London (UK)), Paul G. Williams (Milpark Hospital, Johannesburg (ZA)), Shaun Yo (Department of Respiratory Medicine, The Alfred Hospital, Melbourne, VIC, Australia), Roham T. Zamanian (Division of Pulmonary, Allergy, and Critical Care, and Vera Moulton Wall Center for Pulmonary Vascular Disease, Stanford University (USA)), Zhenguo Zhai(Department of Pulmonary and Critical Care Medicine, Center of Respiratory Medicine, China‐Japan Friendship Hospital, Beijing, China; National Center for Respiratory Medicine, China; State Key Laboratory of Respiratory Health and Multimorbidity, China; National Clinical Research Center for Respiratory Diseases, China; Institute of Respiratory Medicine, Chinese Academy of Medical Sciences, China), Zhu Zhang (Department of Pulmonary and Critical Care Medicine, Center of Respiratory Medicine, China‐Japan Friendship Hospital, Beijing, China; National Center for Respiratory Medicine, China; State Key Laboratory of Respiratory Health and Multimorbidity, China; National Clinical Research Center for Respiratory Diseases, China; Institute of Respiratory Medicine, Chinese Academy of Medical Sciences, China).
