# Supplementary material for: PVRI‐GoDeep—A Global Meta‐Registry at the Crossroads of Heart and Lung
Source: Compr Physiol. 2026 Mar 24;16(2):e70118. doi: 10.1002/cph4.70118 (PMC13013090; doi:10.1002/cph4.70118)
Supplement: Supplementary file 1 — Table S1: Example of equations used for variable conversion and consistency checks. The equations in the table are used for variable conversions and consistency checks, and any necessary rearrangements of these equations are applied. The Du Bois formula is used to calculate body surface area. The BNP to NT‐proBNP conversion is based on data from Rorth et al. (2020), with a total least squares regression model fitted to determine the intercept and slope. [file CPH4-16-e70118-s001.docx]

**Supplement**

**Table S1 Example of equations used for variable conversion and consistency checks.**The equations in the table are used for variable conversions and consistency checks, and any necessary rearrangements of these equations are applied. The Du Bois formula is used to calculate body surface area. The BNP to NT-proBNP conversion is based on data from Rorth et al. (2020), with a total least squares regression model fitted to determine the intercept and slope.

CO = cardiac output; CI = cardiac index; BSA = body surface area; BMI = body mass index; PVRI = pulmonary vascular resistance index; PVR = pulmonary vascular resistance; mPAP = mean pulmonary arterial pressure; PAWP = pulmonary artery wedge pressure; SVR = systemic vascular resistance; mSAP = mean systemic arterial pressure; CVP = central venous pressure; TLC = total lung capacity; VC = vital capacity; RV = residual volume; FEV1 = forced expiratory volume in 1 second; FVC = forced vital capacity; DLCO = diffusing capacity of the lung for carbon monoxide; VA = alveolar volume; BNP = B-type natriuretic peptide; NTproBNP = N-terminal prohormone of brain natriuretic peptide; sPAP = systolic pulmonary artery pressure; dPAP = diastolic pulmonary artery pressure; RVESA = right ventricular end systolic area; RVEDA = right ventricular end diastolic area.

| Equations |
| --- |
| $CO=CI\cdot BSA$ |
| $BMI=weight\cdot\left( \frac{\mathrm{height}}{100} \right)^{-2}$ |
| $BSA=0.007184\cdot height^{0.725}\cdot weight^{0.425}$ |
| $PVRI=PVR\cdot BSA$ |
| $PVR=\frac{mPAP-PAWP}{\mathrm{CO}}$ |
| $SVR=\frac{mSAP-CVP}{\mathrm{CO}}$ |
| $TLC=VC+RV$ |
| $BNP=exp((log(NTproBNP)-0.0786374626846404)/1.34822075061461)$ |
| $mPAP<sPAP$ |
| $mPAP>dPAP$ |
| $RVESA<RVEDA$ |
